# Study of Factors Influencing the Bioaccessibility of Triazolone in Cherry Tomatoes Using a Static SHIME Model

**DOI:** 10.3390/ijerph15050993

**Published:** 2018-05-15

**Authors:** Yu-Ying Liu, Jin-Jing Xiao, Yun-Yao Fu, Min Liao, Hai-Qun Cao, Yan-Hong Shi

**Affiliations:** 1 School of Resource & Environment, Anhui Agricultural University, Hefei 230036, China; yuyingLIU919@163.com; 2 Provincial Key Laboratory for Agri-Food Safety, Anhui Agricultural University, Hefei 230036, China xiaojj187012@163.com (J.-J.X.); liaomin3119@126.com (M.L.); haiquncao@163.com (H.-Q.C.); 3 School of Plant Protection, Anhui Agricultural University, Hefei 230036, China; fyy534472105@163.com

**Keywords:** bioaccessibility, triazolone, cherry tomatoes, Simulator of the Human Intestinal Microbial Ecosystem (SHIME), risk assessment

## Abstract

Estimating the influence of bioaccessibility of pesticide residues in fruits and vegetables on dietary exposure is a challenge for human health risk assessment. This study investigated the bioaccessibility of pesticide residues in cherry tomatoes and contributing factors (digestion time, pH, solid/liquid ratio, and dietary nutrition) using an in vitro test simulating the human gastrointestinal tract. pH had the largest effect on triazolone precipitation in the simulated gastric intestinal juice, which had a significant impact on the bioaccessibility. The bioaccessibility of triazolone in the intestinal stage was slightly higher than that in the stomach stage, owing to bile salts and pancreatic enzymes present in the intestinal juice. The bioaccessibility of triazolone did not change significantly with digestion time. In the gastric stage, there was a logarithmic relationship between the bioaccessibility and solid/liquid ratio (R^2^ = 0.9941). The addition of oil significantly changed the bioaccessibility in the gastrointestinal stage. Protein and dietary fiber only affected bioaccessibility in the stomach stage. Dietary nutrition can reduce the release of pesticides from fruits and vegetables into the stomach, sharply reducing the bioaccessibility, and the dietary exposure of pesticide residues in fruits and vegetables can be properly evaluated.

## 1. Introduction

Raw fruits and vegetables with high nutritional values, including cherry tomatoes, are preferred as plant-based foods [1]. In an effort to reduce yield losses and boost production farmers worldwide use fungicides, such as triazolone, to combat plant diseases [2]. However, anthropogenic activities such as fungicide and insecticide application result in pesticide residue pollution [3]. Pesticide application has been documented to lead to adverse health effects, with most research in this area focused on health risks resulting from pesticide residues in fruits and vegetables [4]. Pesticide residues are either the pesticides or their metabolic products that remain in fresh fruits and raw vegetables after application to crops [5]. Most pesticide residues, particularly on raw fruits and vegetables, exhibit bioaccumulation properties, which could lead to harmful effects on humans [6].

To better understand the constraints of this process, the bioaccessibility of pesticide residues should be examined [7]. Bioaccessibility is a crucial step for allowing contaminants to be bioavailable during the digestion process [8]. It is defined as the ratio of the fraction of contaminant mobilized from the food substrate during gastrointestinal digestion to the total amount of contaminants, enabling the correlation of contaminants in matrixes with the amount actually absorbed and metabolized by the gastric mucosa and intestinal epithelium [9]. Many studies have focused on the oral bioaccessibility of heavy metals [10,11] and other contaminants, e.g., mycotoxins [12] and polycyclic aromatic hydrocarbons (PAHs) [13]. For pesticide residues, a Simulator of the Human Intestinal Microbial Ecosystem (SHIME) method for assessing the dietary exposure of pesticide residue accurately have been adopted and shown to be serviceable, based on our previous work [14,15]. Notably, bioaccessibility has been incorporated into human health risk assessments [16].

Furthermore, studies have shown that a variety of factors can affect the bioaccessibility, including digestion time, pH, and solid–liquid (S/L) ratio [17]. When eating fruits and vegetables, other foods may also be ingested and mixed in the gastrointestinal environment, which could affect the bioaccessibility of pesticide residues in fruits and vegetables [18]. In this case, the addition of dietary nutrients is important in the study of bioaccessibility [19]. Bioaccessibility should be considered an important parameter in evaluating the risk of pesticide residues in fruits and vegetables [20]. However, information available on pesticide bioaccessibility and exposure risk in fruits and vegetables during digestion is limited. Although current research is mostly related to the bioaccessibility of heavy metals in soils and some other foods, it could be applied to a wider range of food items, wildlife species, and active ingredients in the future [21,22,23].

In this study, we used an in-vitro SHIME model combining human gastric and intestinal digestion to: (i) quantify the bioaccessibility of triazolone used on cherry tomatoes; (ii) investigate the influence of gastrointestinal digestion parameters (gastric pH, S/L ratio, and digestion time); and (iii) investigate the effects of dietary components (oil, protein, and dietary fiber) on the bioaccessibility of pesticide residues in cherry tomatoes.

## 2. Materials and Methods

### 2.1. Chemicals and Samples

Ultra resi-analyzed grade methanol and acetonitrile used to prepare standards were supplied by Tedia Company, Inc. (Fairfield, OH, USA). Arabinogalactan, peptone, xylan, pectin, soluble starch, mucin, cysteine, glucose yeast, pepsin, bile extract, and pancreatin were purchased from Meifeng Chemical Industry Co., Ltd. (Deyang, Sichuan, China). Water was purified using a CSR-1-20 ultra-high purity (UHP) water system (Beijing Ace Tektronix Technology Development Co., Ltd., Beijing, China). Anhydrous magnesium sulfate (MgSO_4_), primary secondary amine (PSA), and C_18_ were obtained from Meifeng Chemical Industry Co., Ltd. and Agela Technologies Inc. (Tianjin, China), respectively.

A pesticide standard of triazolone (99.9% purity) was purchased from the National Pesticide Quality Supervision and Inspection Center (Beijing, China). Individual stock standard solutions of the pesticide were prepared by dissolving 100 mg of each compound in 100 mL of methanol. Standard working solutions were prepared daily through appropriate dilution of aliquots of the stock solution with methanol and stored at 4 °C in a refrigerator. Cherry tomatoes were purchased from local markets in Hefei, China. All samples were homogenized and stored at −20 °C until analysis.

### 2.2. In-Vitro Digestion Model Based on Human SHIME

The bioaccessibility of triazolone in cherry tomatoes was modified using the Simulator of the Human Intestinal Microbial Ecosystem (SHIME) with some modifications [24,25]. The simulated gastric solution was a mixture of nutritional medium (200 mL) and artificial gastric acid (25 mL). Nutritional medium (1 L) was prepared from arabinogalactan (1 g), pectin (2 g), xylan (1 g), starch (3 g), glucose (0.4 g), yeast extract (3 g), peptone (1 g), mucin (1 g), and cysteine (0.5 g), which was then autoclaved at 121 °C for 20 min. Artificial gastric acid was composed of pepsin (0.089 g) dissolved in 0.10 M hydrochloric acid solution (1 L). The artificial gastric solution was modified to obtain the intestinal solution by adding NaHCO3 (12.5 g), Pulvis Fellis Suis (PFS) (6 g) and porcine pancreatin (0.9 g).

Briefly, cherry tomatoes (0.5 g) containing triazolone were weighed into an individual centrifuge tube (tube volume, 50 mL) and gastric solution (20 mL) was added. The mixture was shaken on its side at 100 rpm for 1 h to mimic the slow gastric phase of food. The sample then underwent simulated intestinal digestion or chemical analysis. After the stomach simulation, intestinal digestion was simulated by adding intestinal juice (10 mL) to each replicate followed by further shaking for 4 h.

### 2.3. Chemical Analyses

After the SHIMEs had been run, all samples were centrifuged for 5 min at 4000 rpm. To evaluate extraction, supernatant (10 mL) was taken from each centrifuge tube and vortex mixed and extracted with acetonitrile (20 mL) for 2 min. After centrifugation for 5 min, supernatant (5 mL) was transferred into a 15-mL polypropylene centrifuge tube containing a combination of different dispersive solid-phase extraction (d-SPE) adsorbents: PSA (150 mg) + C_18_ (150 mg) + MgSO_4_ (750 mg). The tube was vortexed for 1 min and centrifuged at 3500 rpm for 3 min. Next, supernatant (2 mL) was transferred into a 10-mL glass tube and evaporated to dryness under a stream of nitrogen over a 40 °C water bath. The residue was then redissolved in methanol (1 mL) and filtered through a 0.22-μm PTFE filter prior to HPLC analysis.

Chemical analyses were processed using our previously described method with slight modification [26]. Extracts were analyzed by HPLC with fluorescence detection (excitation at 230 nm). Separation was achieved using a C18 column (Agilent Zorbax 5-mm C_18_ 250 × 4.6 mm). The flow rate was 1 mL/min throughout analysis using 30% water and 70% methanol, and the injection volume was 10 μL. The run time was 15 min, with retention times of 8.9–9.0 min.

### 2.4. Parameters of Bioaccessibility

To explore the influence of bioaccessibility in vegetables and fruits, the roles of digestion time, pH, S/L ratio, and dietary nutrition were investigated for the duration of the SHIME.

The pH value affects the state and environmental behavior of pollutants. The cherry tomato samples were disposed and transferred into the gastric and intestinal juices with pH values adjusted to 1.68, 2.12, 2.62, 3.01, 4.04, 4.97, 6.02, 6.48, 7.01, 7.48, and 8.00.

The S/L ratio is the ratio of intake matrix to simulated digestive juice. The volume of digestive juice was kept constant, while 0.2, 0.4, 0.5, 1.0, and 2.0 g of cherry tomatoes were used in different treatments. Subsequent steps were the same as detailed above.

Based on the physiological conditions of the human gastrointestinal system, the digestion times of the gastric and intestinal stages were set to 30, 60, 90, 120, and 180 min, and 1, 2, 4, 6, and 8 h, respectively.

### 2.5. Effect of Dietary Nutrition on Bioaccessibility

In the human consumption of fruits and vegetables, other foods may be ingested successively and become mixed in the gastrointestinal environment to affect the bioaccessibility of pesticide residues in fruits and vegetables. In accordance with Chinese Dietary reference intakes (DRIs), protein (0.02, 0.05, 0.1, 0.2, and 0.5 g), oil (0.2, 0.4, 0.6, 0.8, and 1 mL), and dietary fiber (0.01, 0.02, 0.05, 0.1, and 0.2 g) were added to the cherry tomatoes samples prepared with triazolone to evaluate the influence of dietary nutrition on bioaccessibility.

### 2.6. Statistical Analysis

Data are expressed as the mean ± standard error (SE). Statistical analysis of each parameter was performed using analysis of variance (ANOVA) followed by Tukey’s test. All figures were drawn using Graphad Prism 7 (GraphPad Software, Inc., San Diego, CA, USA). Differences among means were considered statistically significant at a *p*-value of 0.05. Bioaccessibility of the cherry tomatoes samples (gastric and intestinal) was calculated using the following formula:

$$\mathrm{Bioaccessibility}\;\left(\mathrm{BA},\;\%\right)=\frac{C_{1}\times V}{C_{2}\times M}\times 100\%$$

where C_1_ is the concentration of the compound of interest in the intestinal or gastric juice (mg/kg), V is the volume of intestinal orgastric juice (mL), C_2_ is the concentration of the compound of interest in the cherry tomatoes sample (mg/kg), and M is the weight of the cherry tomatoes sample (g).

## 3. Results and Discussion

### 3.1. Method Validation

A QuEChERS method with high performance liquid chromatography (HPLC) was used to analyze pesticide residues in simulated gastrointestinal digestion. The calibration curve was established by drawing the peak area in comparison with the instrument response and target pesticide concentration. The correlation coefficient (R^2^) of the calibration curves was 0.9996. The limit of quantification (LOQ) was 0.01 mg/L, described as the minimum analyte concentration yielding 10 times the signal-to-noise (S/N) ratio. The figures of merit of the proposed technique are shown in Table 1.

To evaluate the recovery and precision, blank cherry tomato samples were spiked with triazolone at three concentration levels (0.5, 1, and 5 mg/L). Table 1 shows that the recoveries of the added standard were 82.21–109.74%, 90.55–113.73%, and 99.70–103.23% at spiked concentrations of 0.5, 1, and 5 mg/L, respectively. The relative standard deviations (RSDs, %) of quintuplicate experiments for triazolone at each concentration level were below 10%, demonstrating that the developed method was satisfactory for verifying pesticide residues.

### 3.2. Effect of Gastrointestinal pH on Triazolone Bioaccessibility

The pH value is a main factor influencing the bioaccessibility of pollutants [27]. An empty stomach has a high stomach acid content and corresponding low pH value (pH 1.0–2.0), while a full stomach has a slightly higher pH value (pH 4.0–5.0) owing to food dilution and digestive enzymes. The human intestinal environment contains bile, pancreatic juice, and various digestive enzymes, such that the pH value was around 5.5–7.5 [28,29].

**Figure 1:**
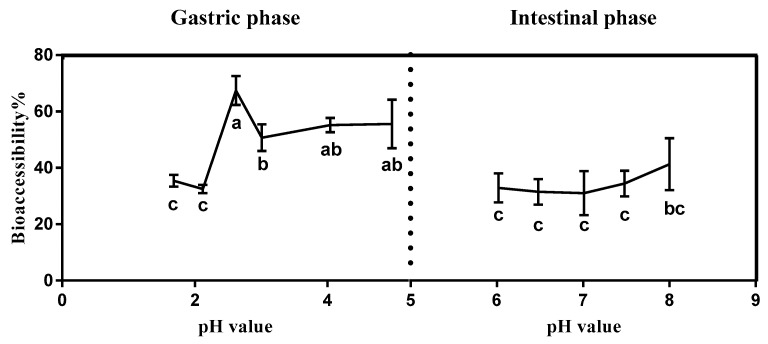
Effects of gastric phase pH and intestinal phase pH on triazolone bioaccessibility. Results are reported as mean ± SE (calculated from three independent experiments). Different minor case letters mean significant differences of essential oil at a *p* value of 0.05.

Figure 1 showed the effect of gastrointestinal pH on the bioaccessibility of triazolone in cherry tomatoes. The bioaccessibility of triazolone was maximized at pH 2.62 in the simulated gastric juice. However, the bioaccessibility did not significantly change in the pH range of 6–8 in the simulated intestinal juice, with the highest bioaccessibility of the intestinal stage (41.33%) reached at pH 8.00. The bioaccessibility of triazolone in cherry tomatoes was generally relatively higher during the stomach stage (32.47–67.40%) than in the intestinal stage (31.00–41.33%), among which the significant difference at pH 2.63 (67.4% of bioaccessibility). This result was similar to those reported in previous related bioaccessibility studies [20]. The bioaccessibility of the intestinal stage was lower than that of the stomach, primarily by virtue of the physicochemical properties of triazolone. It also possible that triazolone showed higher affinity with pepsin compared with pancreatin and subsequently decreased the effect of pH and the release of triazolone in the gastric phase [30]. As shown in Figure 1, the pH was increased from the gastric stage (pH 1.68–4.97) to the intestinal stage (pH 6.02–8.00), which generated a decrease in the bioaccessibility of triazolone. This might be due to triazolone being more stable under alkaline conditions, which resulted in lower bioaccessibility. Furthermore, there was no significant change in bioaccessibility in the intestinal stage, indicating that the properties of triazolone were stable under neutral and alkaline conditions [31].

### 3.3. Effect of Digestion Time on Triazolone Bioaccessibility

Based on the physiological conditions of the human stomach, the digestion time of the gastric stage was approximately 1–3 h, while that of the intestinal stage was 1–18 h [32]. Generally, increasing the gastrointestinal digestion time gradually increases the dissolution of pollutants in the substrate, until the contaminant is completely released into the gastric intestinal juice, while prolonging the digestion time showed no further change [20,33].

**Figure 2:**
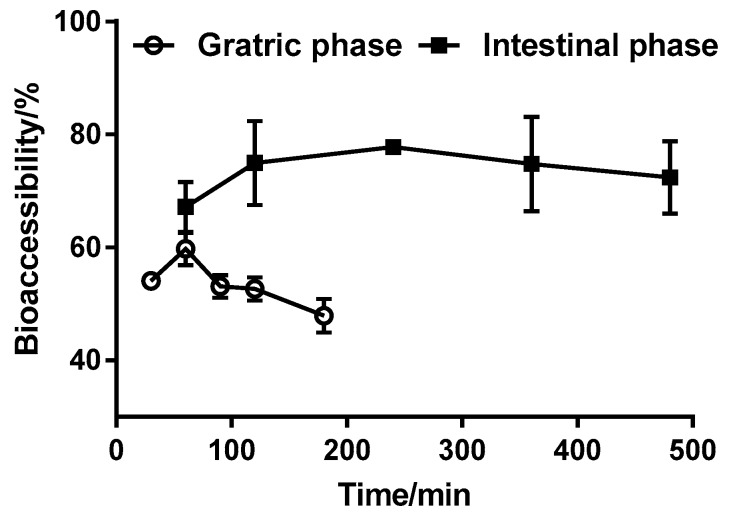
Effects of gastric phase and intestinal phase digestion times on triazolone bioaccessibility. Results are reported as mean ± SE (calculated from three independent experiments).

Figure 2 shows that the bioaccessibility was significantly higher in the simulated intestinal juice than in the simulated gastric juice. Furthermore, the bioaccessibility of triazolone showed no obvious change in the simulated gastric intestinal juice. However, the bioaccessibility showed a slight increase at digestion times of 30–60 min in the stomach stage and 60–240 min in the intestinal stage, and a slight decrease at digestion times of 60–180 min in the stomach stage and 240–480 min in the intestinal stage. Furthermore, bioaccessibility reached maxima of 59.70% and 77.74% at 60 min (stomach) and 240 min (intestinal), respectively. Therefore, as digestion time in the gastrointestinal stage was extended, triazolone was presumably absorbed and bioaccessibility gradually increased until reaching a maximum. As the digestion time was extended further, the bioaccessibility gradually decreased with a modest amplitude. Maldonado-Valderrama et al. [34] found that the presence of bile salts affected the release of heavy metals during the intestinal phase. The biliary salt and pancreatic enzymes in the intestinal juice decreased the interfacial tension of the solution, which had an effect similar to that of a surfactant, resulting in the bioaccessibility of triazolone in the intestinal stage being slightly higher than that in the stomach stage. A similar phenomenon was observed for difenoconazole, hexaconazole, and spirodiclofen in apples, indicating the bile salts have a significant effect on the release of pesticides [15]. This needs further investigation.

### 3.4. Effect of S/L Ratio on Triazolone Bioaccessibility

**Figure 3:**
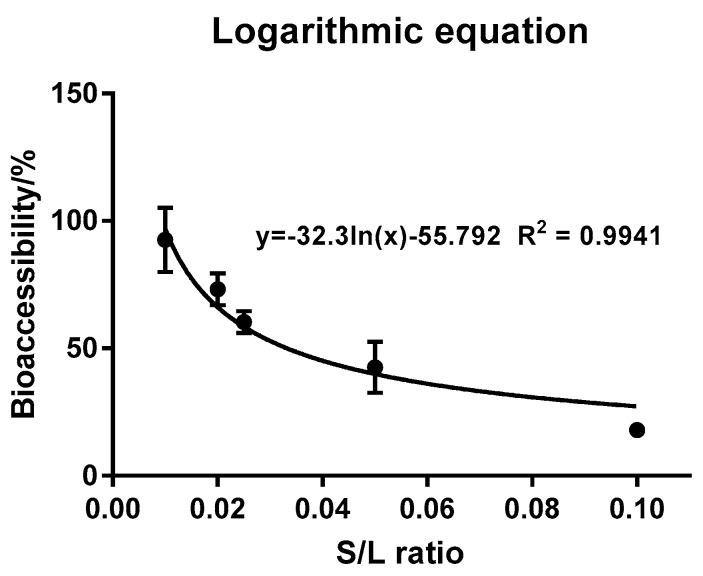
Curve fitting of gastric phase S/L ratio on triazolone bioaccessibility and corresponding correlation coefficients. Results are reported as mean ± SE (calculated from three independent experiments). Fitted curves were plotted using the GraphPad Prism 7 software.

The S/L ratio is the ratio of substrate to simulated digestive juice quantities, which has a large influence on the bioaccessibility of pollutants [35]. The quantity of matrix is determined by the quantity of food ingested, which can be significantly different in practical terms [20]. The S/L ratio was varied in this study by changing the cherry tomato quantity and maintaining the gastric juice volume. As many enzymes and intestinal microorganisms are present in intestinal juice, it was more meaningful to study the effect of S/L ratio on bioaccessibility in the gastric stage. The bioaccessibility of triazolone in the stomach phase was found to be significantly reduced as the S/L ratio relative to stomach fluid increased from 100:1 to 10:1. As the relative quantity of cherry tomatoes was decreased, they were able to become in full contact with the stomach juice, allowing trizolone in the cherry tomatoes to gradually melt into the stomach, which can significantly increase the bioaccessibility. After triazolone was completely dissolved into the simulated gastric juice, continuing to increase the amount of simulated gastric juice showed that the bioaccessibility can be stable within a certain range, which was consistent with the previous studies, e.g., Kang et al. [20] and Smith et al. [36]. Furthermore, the experimental data was fitted in a three-curve fitting relationship to determine the relationship between bioaccessibility and the S/L ratio in vitro. This produced an obvious logarithmic relationship, as shown in Figure 3, with an R^2^ value of 0.9941, suggesting that the simulated relationship was an accurate reflection of the relative bioavailability.

### 3.5. Effect of Dietary Nutrients on Triazolone Bioaccessibility

When eating fruits and vegetables, humans often also ingest other foods, which dramatically affects the bioaccessibility of pesticide residues in fruits and vegetables [37]. The bioaccessibility of DDTs in carrot were found to clearly decrease when spinach, cabbage, and pork were added to the carrot as a mixed food. In particular, spinach and cabbage reduced the bioaccessibility by approximately 78.3–86.1% and 69.8–78.6% respectively [38]. Few other studies on the effects of nutrients on bioaccessibility have been reported. According to Chinese Dietary reference intakes (DRIs), oil, protein, and dietary fiber were added to the simulated gastric intestinal fluid to evaluate the bioaccessibility of triazolone in cherry tomatoes fortified with dietary nutrients, which would provide important information for the scientific assessment food safety of fruits and vegetables.

#### 3.5.1. Effect of Oil on Triazolone Bioaccessibility

**Figure 4:**
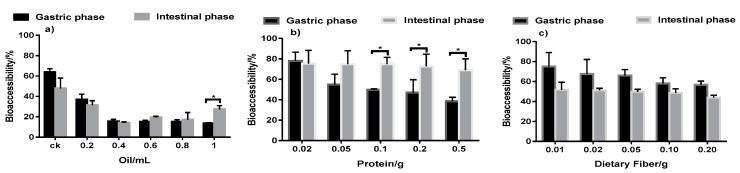
Effects of oil, protein, and dietary fiber in the gastric phase and intestinal phase on triazolone bioaccessibility. Results are reported as mean ± SE (calculated from three independent experiments). CK means that the cherry tomatoes samples were prepared with triazolone but not added oil. The asterisks indicate significant differences in the bioaccessibility (* *p* value < 0.05 and ** *p* value < 0.01).

As shown in Figure 4, the bioaccessibility of triazolone in the gastrointestinal stage decreased significantly with the addition of trace amounts of oil. However, the bioaccessibility tended to become stable after a certain amount of oil was added. In contrast, there was only a slight variation in bioaccessibility in the simulated intestinal fluid, with no significant difference observed between the treatments. The bioaccessibility of triazolone in cherry tomatoes was reduced by nearly 50%. The oil-base surface activation of oil may prevent the migration of pesticides, resulting in the decreased dissolution rate into the gastrointestinal juice [39]. Adding more than 0.4 mL of oil afforded no obvious further change in bioaccessibility, indicating that adding oil did not produce a continuous decrease in bioaccessibility. Therefore, the moderate intake of oil could improve the safety of fruits and vegetables in daily life.

#### 3.5.2. Effect of Protein and Dietary Fiber on Triazolone Bioaccessibility

The effects of dietary fiber and protein on the bioaccessibility of triazolone were similar, as shown in Figure 4. Triazolone bioaccessibility decreased gradually with the addition of dietary fiber and protein in the stomach stage. However, in the intestinal stage, bioaccessibility slightly decreased with the addition of dietary fiber and protein, which might be due to bile powder and pancreatic enzymes in the intestinal juice inhibiting the ability of proteins and cellulose to absorb triazolone [17]. The bioaccessibility in the intestinal stage was less than that in the stomach stage, probably due to dietary fiber enhancing digestion, while protein did not.

## 4. Conclusions

In summary, bioaccessibility is an important parameter when studying the effects of pollutants on organisms and is of great significance in dietary risk assessment. To avoid the high costs and ethical issues involved with in-vivo methods, a modified SHIME model was used to simulate the digestive process of the human gastrointestinal tract. A pesticide residue analysis method based on HPLC was established to analyze triazolone released from cherry tomatoes into the gastrointestinal fluid. The effects of digestion time, pH, S/L ratio, and dietary nutrition on bioaccessibility have been investigated, with the results showing that pH and S/L ratio were the key factors affecting bioaccessibility, while the digestion time also had a non-negligible effect. Furthermore, the effect of oil on bioaccessibility was significant in the both stomach and intestine stages, while protein and dietary fiber only obviously affected bioaccessibility in the gastric stage. This in-vitro gastrointestinal simulation method could be used to correctly assess the risks posed by pesticide residues in fruits and vegetables.

## Figures and Tables

**Table 1:** Analytical recoveries, relative standard deviations (RSDs), correlation coefficient (R^2^) values and limits of quantification (LOQs) for gastrointestinal juice samples studied.

Pesticides	Fortified Level (mg kg^−1^)	Intestinal Juice		Gastric Juice		R^2^	LOQ
		Average Recovery (%)	RSD (%)	Average Recovery (%)	RSD (%)		(mg kg^−1^)
Triazolone	0.5	82.21	2.71	109.74	8.43	0.9996	0.01
	1	90.55	4.08	113.03	5.82		
	5	99.70	1.74	107.23	0.46		
